# Enhanced osmotic dehydration of watermelon rind using honey–sucrose solutions: A study on pre-treatment efficacy and mass transfer kinetics

**DOI:** 10.1515/biol-2022-0946

**Published:** 2024-09-25

**Authors:** Jaspreet Kaur, Sawinder Kaur, Amine Assouguem, Sara El Kadili, Riaz Ullah, Zafar Iqbal, Vikas Nanda

**Affiliations:** Department of Food Engineering and Technology, Sant Longowal Institute of Engineering and Technology, Longowal, 148106, Sangrur, Punjab, India; Department of Agricultural and Food Engineering. Indian Institute of Technology, Kharagpur, West Bengal, India; Department of Food Technology and Nutrition, Lovely Professional University, Phagwara, 144411, Punjab, India; Laboratory of Functional Ecology and Environment, Faculty of Sciences and Technology, Sidi Mohamed Ben Abdellah University, Imouzzer Street, Fez, P.O. Box 2202, Morocco; Department of Plant Protection and Environment, National School of Agriculture, Meknes, Morocco; Department of Animal production, Nationale d’Agriculture de Meknès, Meknes, Morocco; Department of Pharmacognosy College École of Pharmacy King Saud University, Riyadh, Saudi Arabia; Department of Surgery, College of Medicine, King Saud University, P.O.Box 7805, Riyadh, 11472, Kingdom of Saudi Arabia

**Keywords:** watermelon rind, osmotic dehydration, pre-treatments, effective diffusivities, activation energy, overall dehydration coefficient

## Abstract

This study investigates the osmotic dehydration process of watermelon rind using a solution composed of honey and sucrose. The impact of the ratio of rind-to-solution and temperature on the process is illustrated. Pre-treatments such as blanching, microwaves, and ultrasonication were utilized. Ultrasonication reduces the time needed for osmosis in a sample, resulting in increased fluid loss and solute uptake; therefore, it was selected as the method to investigate the kinetics and modelling of mass transfer. The effective diffusivities for water loss (ranging from 3.02 × 10^−5^ to 4.21 × 10^−4^ m^2^ s^−1^) and solid gain (ranging from 1.94 × 10^−6^ to 3.21 × 10^−6^ m^2^ s^−1^) were shown to increase with process variables such as temperature and the rind-to-solution ratio. The activation energy decreased as the process temperature increased, ranging from 3.723 to 0.928 kJ mol^−1^ for water loss and from 1.733 to 0.903 kJ mol^−1^ for solid gain, respectively. The sample treated with microwaves exhibited the maximum dehydration coefficient, rendering it appropriate for producing dehydrated products. Five empirical models were utilized, with the power law model (*R* ^2^ = 0.983) and the Magee model (*R* ^2^ = 0.950) being the most suitable for water loss data and solid gain, respectively.

## 1 Introduction

Watermelon (*Citrullus lanatus*, variety lanatus, family *Cucurbitaceae*) has a high economic value with an annual production estimated to be 93,710 million tonnes. Around 30 million tonnes of watermelon waste are produced as rind and seeds during the production and processing of fruit juices in the food industry [1]. Unfortunately, the disposal of these by-products as waste causes ceaseless difficulties, leading to environmental deterioration. Thus, it is possible to convert this waste into value-added food products. Osmotic dehydration is one such approach. This phenomenon aims to improve product excellence and reliability by altering its physiochemical properties [2]. Osmotic dehydration is governed by the differences in osmotic pressure and concentration gradients [3,4]. Two types of countercurrent flows operate simultaneously when food is engrossed into the osmotic solution: water/moisture discharges from the food into the solution medium, and solute particles from the solution elated into the food [5].

Over the past few decades, osmotic dehydration has gained recognition due to its lower energy consumption, chemical-free preservation of food with higher shelf stability, and better organoleptic properties [6,7,8]. Despite these advantages, the major issue with this process is the prolonged time requirement, which can be overcome using novel techniques such as ultrasonication, microwaves, and high electric field pulses [9,10].

Currently, a plethora of research has demonstrated the combination of the osmotic dehydration process with various other techniques, such as microwave [11], hot-air oven drying [12], ultrasonication [13], and pulse electric field [14], to reduce the extent of food dehydration. This study aimed to provide the most suitable processing method for the development of dehydrated watermelon rind by applying engineering approaches and encouraging the potential of its industrial scale-up. Also, the mass transfer kinetics of waste products, such as watermelon rind, have never been reported before. Additionally, this article presents an innovative approach for comparing various pre-treatments applied to watermelon rinds prior to osmotic dehydration.

## 2 Materials and methods

### 2.1 Raw materials

The century-23 grade variety of watermelon was purchased from the local market of Longowal, Punjab. The fruit was washed and peeled, and the rind was obtained, which was cut into cubes of 5 mm^3^ in size using a knife. Unifloral honey was procured from the Apiary of SLIET, Longowal, Punjab. Food-grade sucrose was purchased from the local market in Longowal. Honey and sucrose were combined, keeping honey constant and varying the sucrose concentration on (weight/volume) basis. The trial experiments were conducted, through which the selected concentration of the osmotic solution was 100:15, in which 100 mL of honey and sucrose were taken as 15% (by weight). The rind-to-solution ratios were selected as 1:1, 1:2, and 1:3 throughout the experiments.

### 2.2 Osmotic dehydration with various pre-treatments

**Figure 1:**
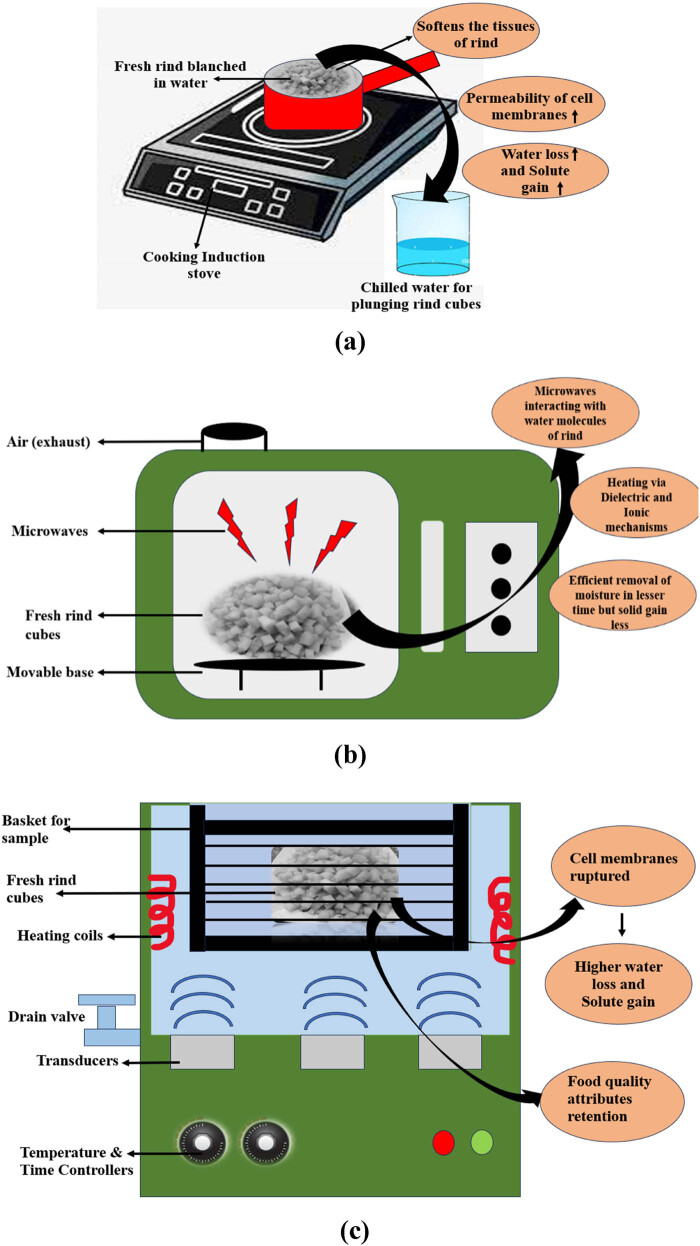
Pictorial presentation of fresh rind subjected to different pre-treatments before the osmotic dehydration process: (a) blanching pre-treatment, (b) microwave pre-treatment, and (c) ultrasonication pre-treatment.

The rind cubes were subjected to three types of treatments: (a) osmotic dehydration with blanching as pre-treatment, (b) ultrasound-assisted osmotic dehydration, and (c) microwave-assisted osmotic dehydration, as shown in Figure 1.

**Figure 2:**
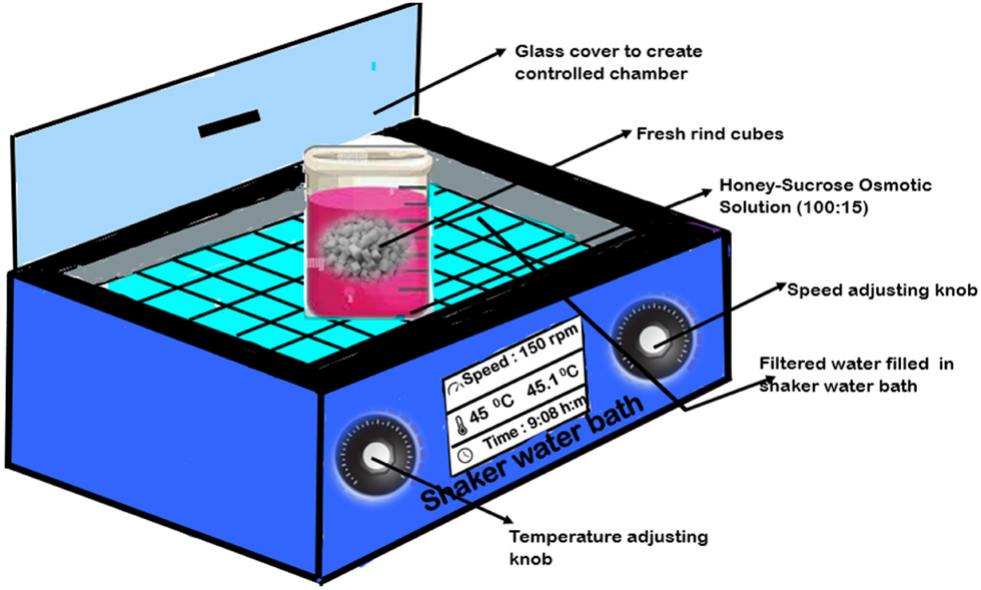
Experimental set-up for osmotic dehydration after subjecting fresh rind to different pre-treatments.

The rind was weighed to 10 g, subjected to blanching at 100°C for 180–240 s, and plunged in cold water. The ultrasonication pre-treatment was given by weighing 10 g of rind cubes and immersing them in 100 mL of distilled water and subjecting them to the ultrasound waves for 1,200 s in the Bath type Ultrasonic equipment (Make Macro Scientific Works; Model MSW 269, New Delhi, India) with a frequency of 25,000 Hz at a constant temperature of 45°C [15]. Furthermore, the rind cubes were subjected to microwave irradiation, generated at 2,450 Hz at 30% of 700 W power for 120 s as a third pre-treatment. Afterward, the cubes were dipped into the honey–sucrose-based osmotic solution (100:15) with varying fruit-to-solution ratios (1:1, 1:2, 1:3), placed in a shaker water bath with agitation at 150 rpm, and set at different temperatures (45, 55, and 65°C) until equilibrium conditions were attained. The cubes were rinsed with distilled water and blotted with tissue paper. The cubes were weighed to determine the amount of water loss and solid gain at regular intervals. The experimental set-up for the osmotic dehydration is illustrated in Figure 2.

### 2.3 Modelling of osmotic dehydration phenomenon

The two processes, water loss and solids gain, occurring simultaneously to and from the fruits, were calculated as

(1) \[\text{Soild}\hspace{.5em}\text{gain}\hspace{.5em}\text{(SG)}\hspace{.5em}\text{=}\hspace{.5em}\text{Soild}\hspace{.5em}\text{gain}\hspace{.5em}(\text{g})/100\hspace{.1em}\text{g}\hspace{.25em}\text{fresh rind}\hspace{.5em}\text{=}\hspace{.5em}\text{(}{m}_{1}-{m}_{2}),]\]

\[\text{Weight reduction}(\text{WR})=({M}_{2}-{M}_{1})]\]

\[\text{Water loss}=\text{SG}+\text{WR}(\text{g})]\]

(2) \[{\mathrm{Water\; loss}}/100\hspace{.5em}{\mathrm{g\; of\; rind\; cubes}}=\hspace{1em}\frac{{\mathrm{WL}}({\mathrm{g}})\hspace{0.25em}\times 100}{{M}_{1}},]\]

(3) \[{\mathrm{Solid\; Gain}}/100\hspace{.5em}{\mathrm{g\; of\; rind\; cubes}}=\hspace{1em}\frac{{\mathrm{SG}}({\mathrm{g}})\times 100}{{M}_{1}}]\]

where weight of rind cubes before osmotic dehydration = *M*_1_ (g), weight of rind cubes after osmotic dehydration at any time (*t*) = *M*_2_ (g), initial dry matter (before osmotic dehydration) = *S* × *M*_1_ = *m*_1_, and final dry matter (after osmotic dehydration) = *S* × *M*_2_ = *m*_2._

#### 2.3.1 Calculation of effective diffusivity of solids and moisture during osmotic dehydration

The following well-known equations for the transfer of water and solute, respectively, are obtained from the solution of Fick’s second law for diffusion from a rectangular parallel-pipe (sides 2a, 2b, and 2c) [16,17].

(4) \[{\mathrm{MR}}=\frac{{M}_{2}-{M}_{{\mathrm{\infty }}}}{{M}_{1}-{M}_{{\mathrm{\infty }}}}=\mathop{\sum }\limits_{n=1}^{{\mathrm{\infty }}}{C}_{n}^{3}\exp \left[-{D}_{{\mathrm{w}}}t{q}_{n}^{2}\times \left(\frac{1}{{a}^{2}}+\frac{1}{{b}^{2}}+\frac{1}{{c}^{2}}\right)\right]]\]

and

(5) \[{\mathrm{SR}}=\frac{{m}_{2}-{m}_{{\mathrm{\infty }}}}{{m}_{1}-{m}_{{\mathrm{\infty }}}}=\mathop{\sum }\limits_{n=1}^{{\mathrm{\infty }}}{C}_{n}^{3}\exp \left[-{D}_{{\mathrm{s}}}t{q}_{n}^{2}\times \left(\frac{1}{{a}^{2}}+\frac{1}{{b}^{2}}+\frac{1}{{c}^{2}}\right)\right],]\]

where MR and SR are moisture and solid ratio, and *M*_1_, *M*_2,_ *m*_1_, and *m*_2_ are the initial and final weights of the rind cubes and the initial and final dry matter, respectively. *M*_∞_ and *m*_∞_ are the weight and dry matter at equilibrium conditions, and *D*_ew_ and *D*_es_ are effective diffusivities of water and solute, respectively.

If we consider the rind as a cubical configuration, then

(6) \[{C}_{n}^{3}=\frac{2\alpha (1\left+\alpha )}{(1+\alpha +{\alpha }^{2}\left+{q}_{n}^{2})},]\]

where \[{q}_{{ns}}^{^{\prime} \hspace{0.25em}}]\] is the non-zero positive roots of the equation ( \[\tan {q}_{{n}}=\left-\alpha {q}_{{n}})]\] and ∝ is the volume of the ratio of the rind to solution.

As all the sides of cubes are equal (*a* = *b* = *c*), equations (1) and (2) are reduced to

(7) \[{\mathrm{MR}}=\frac{{M}_{2}-{M}_{{\mathrm{\infty }}}}{{M}_{1}-{M}_{{\mathrm{\infty }}}}=\mathop{\sum }\limits_{n=1}^{{\mathrm{\infty }}}{C}_{n}^{3}\exp \left[-{D}_{{\mathrm{w}}}t{q}_{n}^{2}\times \left(\frac{3}{{a}^{2}}\right)\right]]\]

and

(8) \[{\mathrm{SR}}=\frac{{m}_{2}-{m}_{{\mathrm{\infty }}}}{{m}_{1}-{m}_{{\mathrm{\infty }}}}=\mathop{\sum }\limits_{n=1}^{{\mathrm{\infty }}}{C}_{n}^{3}\exp \left[-{D}_{{\mathrm{s}}}t{q}_{n}^{2}\times \left(\frac{3}{{a}^{2}}\right)\right].]\]

For diffusion from the cube, the Fourier number for water loss and solid gain is defined as

(9) \[{D}_{{\mathrm{ew}}}\times t\times \left(\frac{3}{{a}^{2}}\right)]\]

and

(10) \[{D}_{{\mathrm{es}}}\times t\times \left(\frac{3}{{a}^{2}}\right).]\]

Equations (7) and (8) are graphically shown by plotting log (MR or SR) against Fourier number, and the slope was obtained as

(11) \[\frac{{\mathrm{d}}(\log {\mathrm{MR}}\left){\mathrm{or}}\left(\mathrm{log\; SR})}{{\mathrm{d}}\left({\mathrm{Fo}})}]\]

for cubical configuration.

The experimental data were fitted to the solution of mass transfer equations:

for moisture loss,

(12) \[-\frac{{\mathrm{d}}m}{{\mathrm{d}}t}={k}_{m}(m-{m}_{\infty }),]\]

and

for a solid gain

(13) \[\frac{{\mathrm{d}}s}{{\mathrm{d}}t}\left={k}_{s}(s\left-{s}_{\infty }),]\]

where *k* is the mass transfer coefficient, and the slopes were obtained as

(14) \[\frac{{\mathrm{d}}(\log {\mathrm{MR}}\left){\mathrm{or}}\left(\mathrm{log\; SR})}{{\mathrm{d}}t}.]\]

On integrating the above equation,

(15) \[\mathrm{ln}\left(\frac{{M}_{2}-{M}_{{\mathrm{\infty }}}}{{M}_{1}-{M}_{{\mathrm{\infty }}}}\right)={-k}_{m}\times t,]\]

and

(16) \[\mathrm{ln}\left(\frac{{m}_{2}-{m}_{{\mathrm{\infty }}}}{{m}_{1}-{m}_{{\mathrm{\infty }}}}\right)={-k}_{s}\times t.]\]

The graph ln MR/SR vs *t* was plotted, and the slopes were obtained as

(17) \[\frac{{\mathrm{d}}}{{\mathrm{d}}t}(\log {\mathrm{MR\; or}}\log {\mathrm{SR}})=\pm \frac{{k}_{{\mathrm{m\; or\; s}}}}{2.3025}.]\]

The effective diffusivity values were calculated from the equation

(18) \[{D}_{{\mathrm{w\; or\; s}}}=\left[\frac{{\mathrm{d}}(\log {\mathrm{MR}}\left){\mathrm{or}}\left(\log {\mathrm{SR}})}{{\mathrm{d}}t}/\frac{{\mathrm{d}}(\log {\mathrm{MR}}\left){\mathrm{or}}\left(\mathrm{log\; SR})}{{\mathrm{d}}\left({\mathrm{Fo}})}\right]\times \left(\frac{{a}^{2}}{3}\right).]\]

#### 2.3.2 Calculation of activation energy for solid gain and water loss

The Arrhenius equation was used to calculate the activation energy as

(19) \[{D}_{{\mathrm{e}}}={D}_{0}\exp {[}-{E}_{{\mathrm{a}}}\left/R(T\left+273.15)],]\]

where \[{D}_{0}]\] refers to the coefficient of diffusion at infinitely high temperatures, *R* is the ideal gas constant (= 8.314 kJ mol^−1^ K^−1^), and \[{E}_{{\mathrm{a}}}]\] is the activation energy (kJ mol^−1^).

According to the method of Crank (1975) [16], the value of the calculated natural logarithm of \[{\mathrm{ln}(D}_{{\mathrm{w}}})]\] or \[{\mathrm{ln}(D}_{{\mathrm{s}}}),]\] that is water and solid diffusivity, is plotted in a graph contrary to the inverse of temperature (K). The slope of this graph provided the value of activation energy [17].

#### 2.3.3 Various empirical models

Various empirical models, such as the Azura model [18], Magee model [19], Peleg model [20], power law model [21], and process model [22], were used for the prediction of the solid gain and water loss kinetics of the phenomena of osmotic dehydration, as shown in Table 1. A nonlinear regression analysis technique was employed to validate these models.

**Table 1:** Empirical models for kinetic modelling of watermelon rind cubes

Model no.	Model name	Model equation
01	Azura model	\[{\mathrm{WL\; or\; SG}}\left=(B\times t\left\times k)\left/(1+k\left\times t)]\]
02	Magee model	\[{\mathrm{WL\; or\; SG}}\left=(B+k\left\times {t}^{1/2})]\]
03	Power law model	\[{\mathrm{WL\; or\; SG}}=B\times {t}^{n}]\]
04	Process model	\[{\mathrm{WL\; or\; SG}}=B\left\times (1\left‒{e}^{(‒k\left\times t)})]\]
05	Peleg model	\[{\mathrm{WL\; or\; SG}}=k1+k2\times t]\]

#### 2.3.4 Suitability of fit of different empirical models

A statistical analysis was performed using non-linear regression analysis using Origin Pro 2017. The coefficient of correlation, *R* ^2^, was one of the key factors to decide which model was appropriate. Correspondingly, the excellence of fit was evaluated using a variety of statistical measures, including reduced chi-square (*χ* ^2^), sum square error (SSE), and root mean square error (RMSE), which measured the mean square of the divergence between the experimental and projected values for the models. The *R* ^2^ value should be larger, and other parameter values should be smaller to fit the suitable fit [23,24,25]. The statistical variables were computed as follows.

*R* ^2^ was considered as the quantity of disparity around the mean, as demonstrated by the model:

(20) \[{R}^{2}=1-\frac{{{\mathrm{SS}}}_{{\mathrm{residual}}}}{{{\mathrm{SS}}}_{{\mathrm{model}}}+{{\mathrm{SS}}}_{{\mathrm{residual}}}},]\]

(21) \[{\chi }^{2}=\mathop{\sum }\limits_{i=1}^{N}\frac{{({\mathrm{Experimental\; value}}\left-{\mathrm{Predicted\; value}})}^{2}}{(N\left-n)}.]\]

The RMSE must be close to zero considering the difference between the experimental and anticipated results.

(22) \[{\mathrm{RMSE}}=\mathop{\sum }\limits_{i=1}^{N}{\left[\frac{{({\mathrm{Experimental\; value}}\left-{\mathrm{Predicted\; value}})}^{2}}{\left(N)}\right]}^{1/2},]\]

(23) \[{\mathrm{SSE}}=\mathop{\sum }\limits_{i=1}^{N}\frac{{({\mathrm{Experimental\; value}}\left-{\mathrm{Predicted\; value}})}^{2}}{\left(N)}.]\]

### 2.4 Results and discussion

#### 2.4.1 Mass transfer kinetics of watermelon rind cubes

##### 2.4.1.1 Effect of pre-treatments on osmotic dehydration

Three types of pre-treatments were applied to rind the cubes before osmotic dehydration. Equilibrium was attained in approximately 36,000 secin-blanched samples. Blanching reduces the duration of osmotic dehydration by rupturing the cellular structure of rind tissues, leading to increased cell membrane permeability. Thus, it facilitates the removal of moisture and solid gain [26].

The microwave pre-treatment occurred for 120 s at a 30% of 700 W power at 2,450 Hz [27]. The osmotic process required 28,800 s to reach equilibrium. Moisture loss occurred faster in microwave-treated samples owing to the mechanism of microwaves, which causes a higher rate of heating, leading to advancement in the inner fluid pressure of rind cubes on the water molecules, thus facilitating osmosis [28]. The process was aggressive at the start of the phenomenon owing to the greater osmotic potential existing between the rind cubes and osmotic solution. However, the solid gain occurred less compared to other pre-treatments, which occurred due to greater loss of moisture, and shrinkage occurred, thus resisting the passage for solid transfer [29]. Similar results were reported by Manzoor et al., in which moisture loss was greater in microwave-assisted osmotic dehydration, whereas the solid gain was much lower than that in conventional osmotic dehydration [30].

Ultrasonication was conducted at a frequency of 25,000 Hz and a constant temperature of 45°C for 1,200 s. According to the data, the highest amounts of water lost and solid gained were 28.185 and 10.024 g, respectively, while the temperature varied between 45 and 65°C. Moreover, when the rind to solution ratio ranged from 1:1 to 1:3, the highest recorded amount of fluid loss was 29.840 g, while the solute gain was 10.657 g. Therefore, the system reached equilibrium after 21,600 s, and there was no additional mass transfer. The primary phenomenon observed during ultrasonication is cavitation [31]. The mechanical stress zone induced by ultrasonic waves passing through a solid/liquid system is distinguished by consecutive increases and decreases in the local pressure, resulting in the boiling of the liquid and the creation of many bubbles with the surrounding air [7]. These cavitation bubbles remained stable until the internal pressure exceeded the surface tension. However, subsequently, the bubbles develop steadily and then undergo a dramatic explosion as the ambient temperature (500 K) and pressure (101.325 × 10^6^) increase [32]. The collapse of the food matrix results in the disintegration of the cellular membrane, allowing for a more efficient release of fluids. The additional impacts of this phenomenon include the production of shear stress, microstreaming of moisture, and formation of free radicals resulting from the fission of water molecules during this severe bombardment [33].

##### 2.4.1.2 Effect of solution temperature on osmotic dehydration

**Figure 3:**
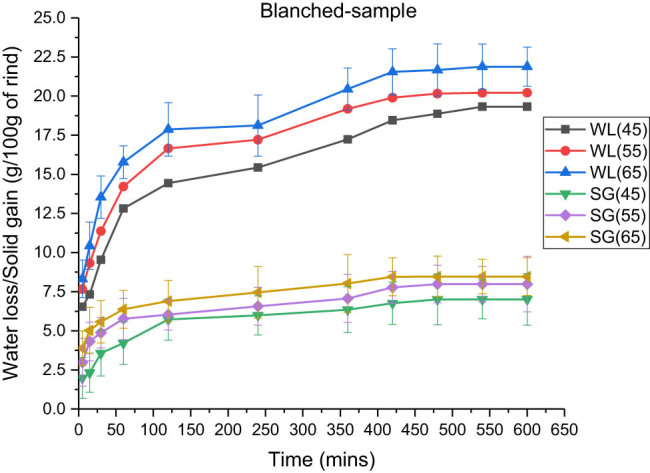
Effect of temperature on water loss and solid gain in the blanched samples at an osmotic solution concentration of 100:15 and a rind-to-solution ratio of 1:3.

**Figure 4:**
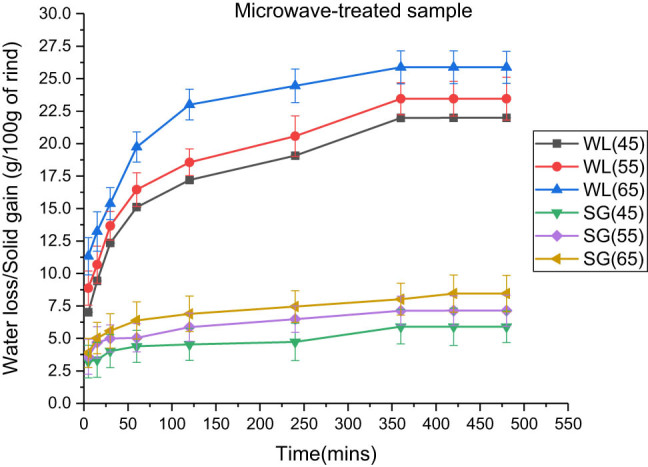
Effect of temperature on water loss and solid gain in the microwave pre-treated samples at an osmotic solution concentration of 100:15 and a fruit-to-solution ratio of 1:3.

**Figure 5:**
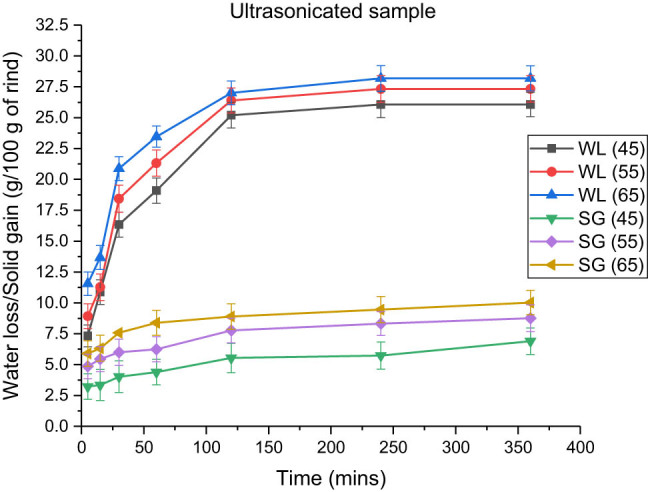
Effect of temperature on water loss and solid gain in ultrasound-treated samples at a rind-to-solution ratio of 1:3 and an osmotic solution concentration of 100:15.

The effects of temperature on water loss and solid gain for various pre-treatments are shown in Tables 2–4. According to data, the maximum water losses that occurred were 21.88 ± 1.25, 25.875 ± 1.22, and 28.185 ± 1.01 g, respectively, for blanched, microwaved, and ultrasonicated samples at 65°C. Furthermore, the solid gains observed were 9.124 ± 1.21, 8.452 ± 1.40, and 10.02 ± 0.98 g, respectively, at 65°C for blanched, microwave-treated, and ultrasonicated rind cubes. Figures 3–5 illustrate that there was an increase in the fluid loss and solid gain, with a significant increase in temperature from 45 to 65°C of the osmotic solution at 100:15 concentration in which the rind cubes were immersed.

**Table 2:** Effect of temperature on water loss and solid gain of blanched watermelon rind cubes

Time (min)	WL (45°C)	WL (55°C)	WL (65°C)	SG (45°C)	SG (55°C)	SG (65°C)
5	6.546 ± 1.21	7.657 ± 1.25	8.321 ± 1.2	1.983 ± 1.31	2.983 ± 1.52	4.435 ± 1.11
15	7.321 ± 1.13	9.325 ± 1.27	10.436 ± 1.51	2.321 ± 1.25	4.321 ± 1.24	5.768 ± 1.47
30	9.546 ± 1.09	11.372 ± 1.26	13.547 ± 1.35	3.567 ± 1.45	4.875 ± 0.98	6.532 ± 1.36
60	12.821 ± 1.08	14.218 ± 1.27	15.782 ± 1.05	4.213 ± 1.36	5.763 ± 1.32	7.657 ± 1.21
120	14.436 ± 1.32	16.659 ± 1.16	17.874 ± 1.71	5.721 ± 1.32	6.032 ± 0.99	8.343 ± 1.32
240	15.433 ± 1.21	17.214 ± 1.18	18.124 ± 1.95	5.989 ± 1.25	6.567 ± 1.21	8.453 ± 1.65
360	17.231 ± 1.22	19.186 ± 1.09	20.453 ± 1.35	6.342 ± 1.47	7.065 ± 1.54	8.768 ± 1.85
420	18.456 ± 1.32	19.898 ± 1.08	21.546 ± 1.47	6.763 ± 1.35	7.773 ± 1.02	8.943 ± 1.21
480	18.876 ± 1.43	20.163 ± 1.24	21.678 ± 1.67	7.012 ± 1.62	7.986 ± 1.2	9.123 ± 1.3
540	19.324 ± 1.54	20.213 ± 1.1	21.879 ± 1.45	7.013 ± 1.24	7.989 ± 1.14	9.124 ± 1.1
600	19.325 ± 1.25	20.215 ± 1.14	21.88 ± 1.25	7.013 ± 1.65	7.990 ± 1.77	9.124 ± 1.21

**Table 3:** Effect of temperature on water loss and solid gain in microwave-treated samples at a fruit-to-solution ratio of 1:3 and an osmotic solution concentration of 100:15

Time (min)	WL (45°C)	WL (55°C)	WL (65°C)	SG (45°C)	SG (55°C)	SG (65°C)
5	7.015 ± 1.32	8.875 ± 1.31	11.325 ± 1.26	3.221 ± 1.43	3.589 ± 1.34	3.892 ± 1.12
15	9.423 ± 1.21	10.676 ± 1.42	13.235 ± 1.34	3.35 ± 1.52	4.687 ± 1.21	5.021 ± 1.21
30	12.342 ± 1.09	13.667 ± 1.10	15.382 ± 1.27	4.021 ± 1.23	4.993 ± 1.04	5.582 ± 1.32
60	15.098 ± 1.00	16.456 ± 1.29	19.737 ± 1.23	4.391 ± 1.16	5.045 ± 1.09	6.383 ± 1.43
120	17.201 ± 1.21	18.556 ± 1.02	22.999 ± 1.23	4.535 ± 1.18	6.56 ± 1.08	6.896 ± 1.35
240	19.062 ± 1.32	20.567 ± 1.56	24.452 ± 1.43	4.731 ± 1.29	6.983 ± 1.02	7.455 ± 1.22
360	21.982 ± 1.54	23.456 ± 1.23	25.873 ± 1.32	5.898 ± 1.26	7.134 ± 1.11	8.024 ± 1.23
420	21.983 ± 1.43	23.457 ± 1.34	25.875 ± 1.43	5.899 ± 1.25	7.135 ± 1.15	8.452 ± 1.43
480	21.983 ± 1.04	23.457 ± 1.65	25.875 ± 1.22	5.90 ± 1.23	7.135 ± 1.21	8.452 ± 1.40

**Table 4:** Effect of temperature on water loss and solid gain in ultrasound-treated samples at a fruit-to-solution ratio of 1:3 and an osmotic solution concentration of 100:15

Time (min)	WL (45°C)	WL (55°C)	WL (65°C)	SG (45°C)	SG (55°C)	SG (65°C)
5	7.325 ± 0.89	8.923 ± 0.998	11.547 ± 0.95	3.221 ± 1.03	4.854 ± 0.99	5.892 ± 1.03
15	10.876 ± 0.99	11.272 ± 1.074	13.675 ± 0.97	3.35 ± 1.25	5.452 ± 1.02	6.321 ± 1.05
30	16.342 ± 1.01	18.436 ± 1.097	20.874 ± 0.96	4.021 ± 1.28	5.998 ± 1.06	7.582 ± 12.05
60	19.098 ± 1.02	21.321 ± 1.065	23.464 ± 0.85	4.391 ± 1.03	6.231 ± 1.02	8.383 ± 1.02
120	25.201 ± 1.03	26.392 ± 1.014	27.008 ± 0.95	5.535 ± 1.18	7.768 ± 0.99	8.896 ± 1.03
240	26.062 ± 1.06	27.321 ± 1.08	28.184 ± 1.03	5.731 ± 1.09	8.324 ± 0.96	9.455 ± 1.05
360	26.063 ± 0.99	27.325 ± 1.06	28.185 ± 1.01	6.898 ± 1.06	8.762 ± 1.08	10.02 ± 0.98

Roy et al. (2021) observed similar results when osmotic dehydration was performed in Satkara fruits. A significant increase in temperature caused a decrease in the viscosity of the osmotic solution, which consequently facilitated the diffusion of solutes into the rind cubes, enhancing the permeability of cells [34,35,36,37].

##### 2.4.1.3 Effect of fruit-to-solution ratio

The effect of the rind-to-solution ratio in blanched samples is demonstrated in Table 5, in which the water loss and solid gain were 20.34 ± 1.014 and 9.934 ± 1.052 g, respectively. This amount was highest when the rind-to-solution ratio was 1:3 at 65°C. Similarly, in the microwaved and ultrasonicated rind cubes, the maximum water loss and solid gain were observed to be the greatest at 1:3, followed by 1:2 and 1:1. Similarly, Tables 6 and 7 show that the amounts of water loss in microwaved and ultrasonicated samples are 28.219 ± 1.032 and 29.840 ± 1.024 g and that for solid gain are 7.543 ± 0.968 and 10.657 ± 1.018 g, respectively. Similar results were observed in a study by Thatayaone et al. (2023) on the effects of osmotic agents and concentration on the physical attributes of intermediate-moisture (IM) bananas [38].

**Table 5:** Effect of rind-to-solution ratio on water loss and solid gain in blanched samples immersed in a 100:15 honey–sucrose osmotic solution at a temperature of 65°C

Time (min)	WL (1:1)	WL (1:2)	WL (1:3)	SG (1:1)	SG (1:2)	SG (1:3)
5	4.037 ± 0.998	4.657 ± 1.025	5.214 ± 1.025	2.215 ± 0.987	2.654 ± 1.025	2.987 ± 1.001
15	6.244 ± 0.969	7.219 ± 1.032	7.985 ± 1.014	3.654 ± 0.958	4.358 ± 1.051	4.657 ± 0.982
30	7.743 ± 0.968	8.957 ± 1.065	9.324 ± 1.02	5.987 ± 0.964	6.329 ± 1.062	6.974 ± 0.998
60	9.658 ± 1.098	10.018 ± 1.025	11.659 ± 1.035	6.189 ± 0.967	6.957 ± 1.025	7.326 ± 1.021
120	10.324 ± 1.056	11.871 ± 1.065	13.657 ± 1.025	7.324 ± 1.025	7.987 ± 1.05	8.557 ± 1.025
240	11.354 ± 1.014	13.354 ± 1.025	15.874 ± 1.052	7.654 ± 1.032	8.328 ± 1.05	9.257 ± 1.032
360	13.982 ± 1.025	15.249 ± 0.978	16.985 ± 1.032	8.168 ± 1.021	8.698 ± 1.021	9.354 ± 1.025
420	14.124 ± 1.041	15.896 ± 0.989	18.0257 ± 1.025	8.235 ± 1.02	9.218 ± 1.031	9.575 ± 1.054
480	15.325 ± 1.021	16.125 ± 1.021	20.328 ± 1.051	8.281 ± 1.031	9.587 ± 1.024	9.932 ± 1.032
540	15.326 ± 1.052	16.126 ± 1.025	20.33 ± 1.025	8.285 ± 1.054	9.588 ± 1.032	9.933 ± 1.021
600	15.328 ± 1.021	16.127 ± 1.035	20.34 ± 1.014	8.300 ± 1.025	9.589 ± 1.001	9.934 ± 1.052

**Table 6:** Effect of rind-to-solution ratio on the water loss and solid gain in microwave-treated samples at 65°C immersed in a 100:15 honey–sucrose solution

Time (min)	WL (1:1)	WL (1:2)	WL (1:3)	SG (1:1)	SG (1:2)	SG (1:3)
5	6.015 ± 0.974	6.123 ± 1.051	6.321 ± 1.051	1.897 ± 0.987	2.123 ± 0.989	2.214 ± 0.969
15	8.423 ± 0.987	9.272 ± 1.032	10.342 ± 1.06	2.543 ± 0.994	3.329 ± 0.967	4.436 ± 0.959
30	14.342 ± 0.968	16.436 ± 1.024	18.453 ± 1.035	3.651 ± 1.014	4.874 ± 1.018	5.541 ± 0.947
60	16.098 ± 0.935	18.321 ± 0.998	20.123 ± 1.028	4.872 ± 1.002	5.621 ± 1.091	6.762 ± 0.897
120	18.201 ± 1.01	20.392 ± 0.987	22.342 ± 1.047	5.741 ± 1.034	6.572 ± 0.992	7.246 ± 1.012
240	20.062 ± 1.04	22.321 ± 0.969	24.125 ± 1.02	6.381 ± 0.997	7.032 ± 0.938	7.457 ± 1.045
360	22.982 ± 0.988	26.348 ± 1.012	27.11 ± 0.989	6.677 ± 0.998	7.073 ± 0.994	7.542 ± 1.104
420	23.798 ± 1.021	26.56 ± 1.321	28.218 ± 1.024	6.678 ± 1.012	7.261 ± 1.087	7.543 ± 1.098
480	24.023 ± 0.98	26.61 ± 1.021	28.219 ± 1.032	6.678 ± 1.021	7.262 ± 1.037	7.543 ± 0.968

**Table 7:** Effect of fruit-to-solution ratio for water loss and solid gain in ultrasound-treated samples

Time (min)	WL (1:1)	WL (1:2)	WL (1:3)	SG (1:1)	SG (1:2)	SG (1:3)
5	6.02 ± 1.4	6.234 ± 1.014	6.327 ± 1.021	4.567 ± 0.975	4.598 ± 1.21	5.21 ± 1.054
15	10.123 ± 1.031	11.213 ± 1.021	12.125 ± 1.034	5.432 ± 1.054	6.76 ± 1.014	7.651 ± 1.025
30	15.431 ± 0.912	17.654 ± 0.999	18.201 ± 1.301	6.762 ± 0.998	7.726 ± 1.101	8.874 ± 1.03
60	18.653 ± 0.998	23.324 ± 1.017	25.100 ± 1.038	7.876 ± 0.932	8.981 ± 1.02	9.872 ± 1.214
120	24.564 ± 0.968	26.657 ± 0.997	28.191 ± 1.041	8.864 ± 1.034	9.564 ± 1.2	10.542 ± 1.206
240	25.576 ± 1.01	27.125 ± 1.021	29.839 ± 1.131	9.267 ± 1.047	9.999 ± 1.035	10.654 ± 1.145
360	25.577 ± 0.997	27.125 ± 1.031	29.840 ± 1.024	9.267 ± 1.035	10.012 ± 1.062	10.657 ± 1.018

**Figure 6:**
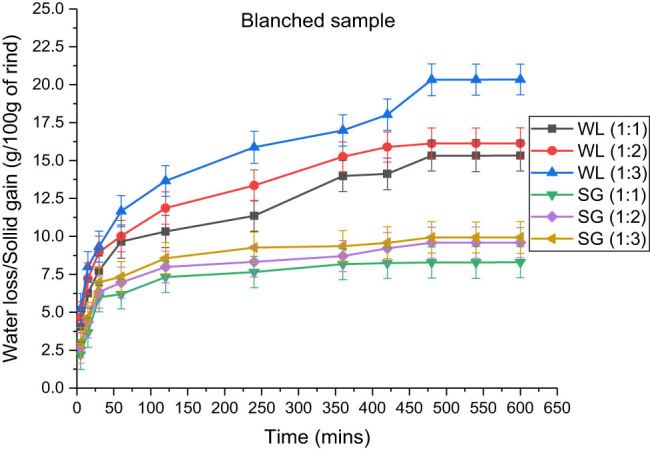
Effect of fruit-to-solution ratio on water loss and solid gain in blanched sample immersed in 100:15 honey–sucrose solution at 65°C.

**Figure 7:**
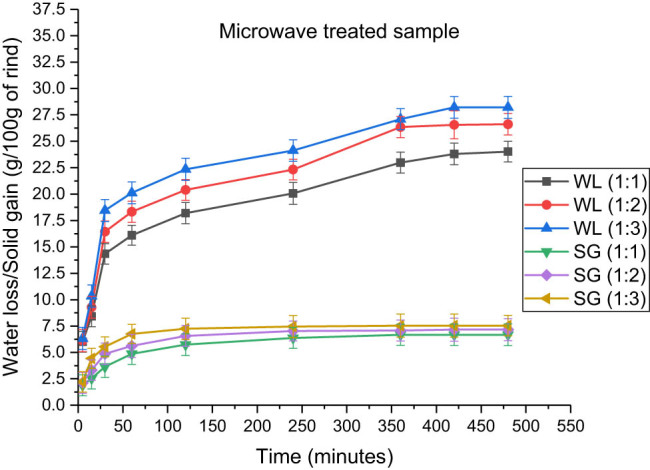
Effect of fruit-to-solution ratio on water loss and solid gain in microwave-treated samples immersed in a 100:15 honey–sucrose solution at 65°C.

**Figure 8:**
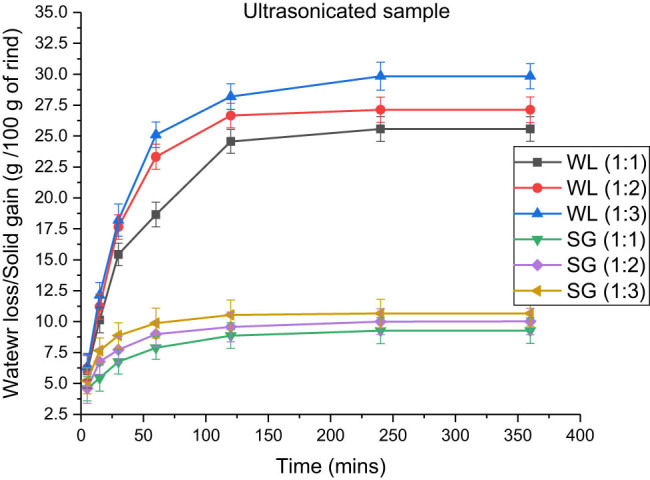
Effect of the rind-to-solution ratio on water loss and solid gain in ultrasound-treated samples immersed in a 100:15 honey–sucrose solution at 65°C.

In addition, Figures 6–8 support the data by depicting the increase in the amount of loss of water and gain of solutes with an increase in the rind-to-solution ratio from 1:1 to 1:3. Similar results were obtained by various authors in their studies [37,39,40], who found that the maximum amount of solute gain and water loss were obtained at high concentrations of osmotic solution. The major reason for this is the increase in the diffusivity of water, along with the reduction in the activation energy due to the increased concentration. Moreover, due to the greater osmotic pressure and the potential difference between the solution outside and water inside, the fluids and solids diffusion became faster, thus providing larger spaces in the cells available for solutes to get occupied [41,42].

#### 2.4.2 Comparative study between different pre-treatments applied to rind cubes

**Figure 9:**
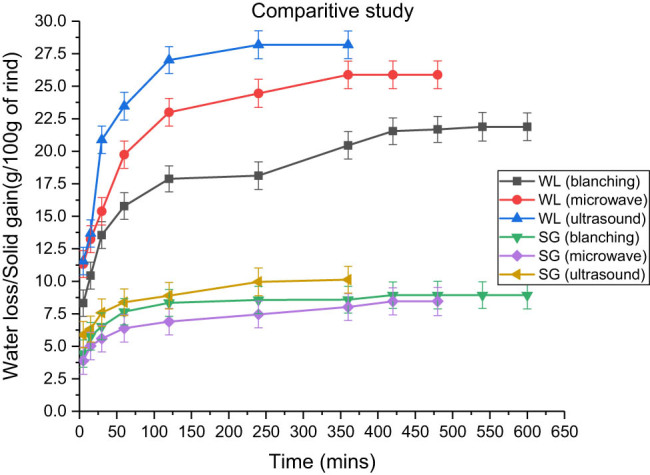
Comparative study between pre-treatments applied to the rind cubes subjected to osmotic dehydration at a rind-to-solution ratio of 1:3 immersed in a 100:15 honey–sucrose solution at 65°C.

Pre-treatments, such as blanching, microwave, and ultrasonication were applied to the fresh rind cubes, and the osmotic dehydration process was performed. Figure 9 illustrates that the duration for osmotic dehydration varied greatly for pre-treated samples. The maximum fluid loss was observed in the ultrasonicated treatment (28.125 ± 1.065 g), followed by the microwave sample (25.185 ± 1.065 g) and blanched sample (21.88 ± 1.068 g), as shown in Table 8. Meanwhile, the solid gain was 10.12 ± 1.064, 8.934, and 8.452 ± 1.081 g in ultrasonicated, blanched, and microwaved samples, respectively. Similar findings were found by Sharif et al. [43] and Singla and Sit [44] in the microwave-assisted drying of sweet potatoes. This contradicting trend of solid gain occurred owing to uneven heating in the rind cubes, which caused more fluid loss from the cells. Eventually, the surface mass transfer resistance disrupts the cell membrane concentration potential, causing a reduction in the solid gain [30,44].

**Table 8:** Comparison between various pre-treatments applied to rind cubes when immersed in a 100:15 concentration osmotic solution at a ratio of 1:3 at 65°C

Time (min)	WL (B) (g/100 g rind)	SG (B) (g/100 g rind)	WL/SG (B)	WL (M) (g/100 g rind)	SG (M) (g/100 g rind)	WL/SG (M)	WL (U) (g/100 g rind)	SG (U) (g/100 g rind)	WL/SG (U)
5	8.321 ± 1.021	4.435 ± 1.052	1.876 ± 1.01	11.325 ± 1.024	3.892 ± 1.052	2.909 ± 1.032	11.547 ± 1.058	5.892 ± 1.014	1.959 ± 1.028
15	10.436 ± 1.035	5.768 ± 1.057	1.809 ± 1.03	13.235 ± 1.034	5.021 ± 1.054	2.635 ± 1.025	13.675 ± 1.052	6.321 ± 0.995	2.163 ± 1.034
30	13.547 ± 1.025	6.532 ± 1.035	2.073 ± 1.021	15.382 ± 1.062	5.582 ± 1.024	2.755 ± 1.051	20.874 ± 1.054	7.582 ± 1.054	2.753 ± 1.037
60	15.782 ± 1.031	7.657 ± 1.014	2.061 ± 1.030	19.737 ± 1.052	6.383 ± 1.052	3.092 ± 1.017	23.464 ± 1.065	8.383 ± 1.029	2.798 ± 1.068
120	17.874 ± 0.998	8.343 ± 1.032	2.142 ± 1.042	22.999 ± 1.063	6.896 ± 1.014	3.335 ± 1.061	27.008 ± 1.035	8.896 ± 1.035	3.035 ± 1.001
240	18.124 ± 1.065	8.564 ± 1.052	2.116 ± 1.025	24.452 ± 1.082	7.455 ± 1.02	3.279 ± 1.035	28.184 ± 1.08	9.955 ± 1.021	2.831 ± 1.095
360	20.453 ± 1.047	8.583 ± 1.024	2.382 ± 1.031	25.873 ± 1.057	8.024 ± 1.028	3.224 ± 1.062	28.185 ± 1.065	10.12 ± 1.064	2.785 ± 1.021
420	21.546 ± 1.028	8.932 ± 1.0241	2.412 ± 1.021	25.875 ± 1.058	8.452 ± 1.045	3.261 ± 1.023			
480	21.678 ± 0.998	8.933 ± 1.062	2.426 ± 1.029	25.875 ± 1.065	8.452 ± 1.081	3.2611.027			
540	21.879 ± 1.097	8.934 ± 1.032	2.442 ± 1.031						
600	21.88 ± 1.068	8.934 ± 1.054	2.449 ± 1.015						

B = blanching pre-treatment, M = microwave pre-treatment, U = ultrasonication pre-treatment, and WL/SG = overall dehydration coefficient.

The overall dehydration coefficient (WL/SG) was also measured for the pre-treated samples, as shown in Table 8. The WL/SG ratio was found to be the highest in the microwave-treated samples, which is desirable for developing dehydrated food products (Sharif et al. [43]). Additionally, there is great potential for developing intermediate moisture food (IMF) products utilizing ultrasonicated samples with an overall dehydration coefficient of 2.785 ± 1.021 g. Similar results were observed by the overall dehydration coefficient was greater in microwave-treated samples [38,45]. This occurred because of the variation in the dielectric characteristics due to the microwave treatment, which led to extra loss of fluid with increased mass transfer [45].

#### 2.4.3 Modelling of the osmotic dehydration process

The moisture and solid diffusivities of ultrasound-assisted osmotic dehydration of rind cubes were kinetically studied. The water loss and solid gain responses were evaluated using Fick’s second law of diffusion’s first Fourier term. The presumption was that all terms except the first were improper [9,46].

Table 9 demonstrates that the watermelon rind cubes gained more solutes and lost more water. Thus, their diffusivities increased due to water loss and solid uptake. The effective diffusivities were calculated from the slope of the natural logarithm of the moisture ratio (MR) or solid ratio (SR) as a function of the dipping time. The values varied from 3.02 × 10^−5^ to 4.21 × 10^−4^ for water loss, and the values for diffusivities varied from 1.94 × 10^−6^ to 3.21 × 10^−6^ for solids. The variation in diffusivities is dependent on various process parameters and conditions.

**Table 9:** Diffusivity and activation energy for moisture loss and solid gain during osmotic dehydration of ultrasound-assisted osmotic dehydration of rind cubes at a 100:15 osmotic solution

F:S (w/w)	Water loss diffusivity ( \[{{\mathrm{m}}}^{2}]\] s^−1^)			Solid gain diffusivity ( \[{{\mathrm{m}}}^{2}]\] s^−1^)			Activation energy (*E*_a_) (kJ mol^−1^) Water loss	Activation energy (*E*_a_) (kJ mol^−1^) Solid gain
	Solution temperature (°C)			Solution temperature (°C)				
	45	55	65	45	55	65		
1:1	3.02 × 10^−5^	3.15 × 10^−5^	3.70 × 10^−5^	1.94 × 10^−6^	2.06 × 10^−6^	2.85 × 10^−6^	3.743	1.733
1:2	5.59 × 10^−5^	5.59 × 10^−5^	6.04 × 10^−5^	2.38 × 10^−6^	2.39 × 10^−6^	2.76 × 10^−6^	1.720	1.359
1:3	3.55 × 10^−4^	3.62 × 10^−4^	4.21 × 10^−4^	2.93 × 10^−6^	3.12 × 10^−6^	3.21 × 10^−6^	0.928	0.903

It was observed that with an increase in the temperature of the osmotic solution at a 100:15 concentration, the diffusivities varied significantly. When the rind-to-solution ratio was increased from 1:1 to 1:3, the effective diffusivities for solute gain varied from 3.02 × 10^−5^ to 3.55 × 10^−4^ for the solution temperature at 45°C. Similarly, it varied from 2.06 × 10^−6^ to 3.12 × 10^−6^ at 55°C and 2.85 × 10^−6^ to 3.21 × 10^−6^ at 65°C. Additionally, the variation in diffusivity occurred for water loss, with an increase in the rind-to-solution ratio from 2.073 × 10^−5^ to 3.55 × 10^−4^ at 45°C, 3.15 × 10^−5^ to 3.62 × 10^−6^ at 55°C, and 3.70 × 10^−5^ to 4.2 × 10^−4^ at 65°C. Similar findings were obtained by Sabetghadam et al. (2024), where the diffusivities increased with variations in the solution temperature and concentration of the osmotic solution [41]. This could be because, as the process parameters varied there were changes in the physical characteristics of the fruit cells, such as enhanced permeability of the cell membranes, which leads to the porous structure, thus facilitating the process [47].

#### 2.4.4 Activation energy for moisture loss and solid diffusivities of watermelon-rind cubes

The activation energy is the minimum energy required for the removal of moisture and intake of solids in rind cubes when soaked in osmotic solutions with varied sucrose concentrations at different temperatures ranging from 45°C to 65°C. The Arrhenius equation was employed for determining the activation energy:

(24) \[\mathrm{ln}D=\mathrm{ln}{D}_{{\mathrm{e}}}+\left[\frac{-{E}_{{\mathrm{a}}}}{R(T\left+273.15)}\right].]\]

The natural log of diffusivity for water or solids was plotted against the inverse of temperature, as a straight line, and the slope of the graph depicts the value of activation energy. According to Table 9, the different values of diffusivities along with the activation energy indicated that the activation energy decreased as the processing conditions varied from 1.733 to 0.903 kJ mol^−1^, with an increase in the solution temperature and rind-to-solution ratio for a solid gain. Similarly, the activation energy for fluid loss varied from 3.743 to 0.928 kJ mol^−1^.

#### 2.4.5 Validation of various empirical models employed for osmotic dehydration

The empirical models were applied to the experimental data under various process conditions and parameters; those with high *R* ^2^ values and lower chi-square (*χ* ^2^), RMSE, and SSE values were accepted. Table 10 shows the values of *R* ^2^, *χ* ^2^, RMSE, and SSE for different models and indicates that all the selected models had coefficients of correlation (*R* ^2^) values greater than 0.90 for both water loss and solid gain at varied temperatures and fruit to osmotic solution ratios (F:S). The two models, Magee and power law models, were best suited for both solid gain and water loss, respectively. The Magee model had *R* ^2^, chi-square, RMSE, and SSE values of 0.950, 0.243, 0.377, and 10.021, respectively, for solid gain, and 0.979, 1.257, 1.116, and 12.775, respectively, for water loss. The power law model resulted in 0.939, 2.242, 0.582, and 4.258 values of the coefficient of correlation, reduced chi-square, RMSE, and sum square error (SSE), respectively, for solid gain, and 0.983, 1.101, 0.978, and 10.056, respectively, for water loss.

**Table 10:** Statistical values of selected models for water loss and solid gain in ultrasonicated samples

Model	F:S	Temperature	Constants (*k*, *n*)		*χ* ^2^	*R* ^2^	RMSE	SSE
Magee model (solid gain)	1:1	45	2.937 ± 0.147	0.149 ± 0.008	0.060	0.967	0.245	0.603
		55	3.576 ± 0.207	0.186 ± 0.012	0.118	0.959	0.344	1.187
		65	2.937 ± 0.147	0.149 ± 0.008	0.060	0.967	0.245	0.603
	1:2	45	3.729 ± 0.152	0.176 ± 0.008	0.064	0.975	0.253	0.644
		55	4.735 ± 0.321	0.275 ± 0.018	0.286	0.955	0.535	2.862
		65	5.392 ± 0.291	0.354 ± 0.017	0.235	0.977	0.484	2.350
	1:3	45	3.913 ± 0.271	0.250 ± 0.015	0.203	0.961	0.450	2.030
		55	4.137 ± 0.668	0.412 ± 0.038	1.233	0.918	1.110	12.33
		65	5.579 ± 0.562	0.456 ± 0.032	0.875	0.950	0.935	8.753
Average			4.103 ± 0.307	0.267 ± 0.017	0.243	0.950	0.377	10.021
Power loss (water loss)	1:1	45	5.53 ± 0.42	0.25 ± 0.01	0.929	0.98	0.963	9.291
		55	5.82 ± 0.33	0.25 ± 0.01	0.58	0.99	0.766	5.867
		65	1.33 ± 0.14	0.44 ± 0.02	0.368	0.99	0.607	3.684
	1:2	45	5.94 ± 0.43	0.23 ± 0.01	0.864	0.98	0.929	8.643
		55	5.90 ± 0.33	0.25 ± 0.01	0.54	0.99	0.736	5.422
		65	7.45 ± 0.38	0.22 ± 0.01	0.59	0.99	0.771	5.985
	1:3	45	6.39 ± 0.67	0.25 ± 0.02	2.275	0.96	1.508	22.75
		55	8.56 ± 0.32	0.21 ± 0.01	0.399	0.99	0.631	3.992
		65	10.42 ± 0.56	0.18 ± 0.01	1.014	0.98	1.007	10.14
Average			6.462 ± 0.51	0.24 ± 0.01	1.101	0.983	0.978	10.056

The Magee model for a solid gain, despite its lower value of *R* ^2^, had a lower SSE value (10.021) compared to that of the power law model. The Azura and process models were rejected due to lower *R* ^2^ and higher Chi-square, RMSE, and SSE values. Similar results were observed by Allahdad et al. (2019) during the ultrasonication-assisted osmotic dehydration of pomegranate arils, in which the Magee model was best suited for solid gain.

## 3 Conclusions

The current research focused on the exploitation of watermelon waste products (rind), which underwent several pre-treatments, such as blanching, ultrasonication, and microwaving. We used the concept of food waste valorization as a sustainable strategy to decrease the amount of trash produced by fruit processing enterprises. Ultrasonication, in combination with osmotic dehydration, is the most suitable method for producing dehydrated food. Therefore, this method may be used effectively with the possibility of scaling up the production capacity to create osmotically dehydrated items. The developed watermelon rind cubes possess a market presence equivalent to confectioneries, serving as snack items, such as candies, enriched with the benefits of honey. Future research should focus on enhancing the nutritional qualities of osmotically dehydrated rind cubes by enriching the osmotic solution with other food waste that is high in bioactive components. Additionally, it is possible to investigate alternative non-empirical models such as mathematical and computational approaches. Additionally, we investigated the various components that influence the osmotic dehydration process. The kinetics of applying hurdle technologies such as freezing, vacuum drying, innovative non-thermal procedures, like cold plasma and pulsed electric fields, and osmotic dehydration can be further investigated.
